# WellExplorer: an integrative resource linking hydraulic fracturing chemicals with hormonal pathways and geographic location

**DOI:** 10.1093/database/baaa053

**Published:** 2020-07-23

**Authors:** Owen Wetherbee, Jessica R Meeker, Caroline DeVoto, Trevor M Penning, Jason H Moore, Mary Regina Boland

**Affiliations:** 1 Department of Biostatistics, Epidemiology and Informatics, Perelman School of Medicine, University of Pennsylvania, Philadelphia, PA, USA, 19104; 2 Institute for Biomedical Informatics, University of Pennsylvania, Philadelphia, PA, USA, 19104; 3 Center for Excellence in Environmental Toxicology, University of Pennsylvania, Philadelphia, PA, USA, 19104; 4 Department of Biomedical and Health Informatics, Children’s Hospital of Philadelphia, PA, USA 19104; 5 Department of Systems Pharmacology & Translational Therapeutics, University of Pennsylvania, Philadelphia, PA, USA

## Abstract

Exposure to hydraulic fracturing fluid in drinking water increases the risk of many adverse health outcomes. Unfortunately, most individuals and researchers are unaware of the health risks posed by a particular well due to the diversity of chemical ingredients used across sites. We constructed WellExplorer (http://WellExplorer.org), an interactive tool for researchers and community members to use for retrieving information regarding the hormonal, testosterone and estrogen modulators located at each well. We found that wells in Alabama use a disproportionately high number of ingredients targeting estrogen pathways, while Illinois, Ohio and Pennsylvania use a disproportionately high number of ingredients targeting testosterone pathways. Researchers can utilize WellExplorer to study health outcomes related to exposure to fracturing chemicals in their population-based cohorts. Community members can use this resource to search their home or work locations (e.g. town or zip code) to determine proximity between where they live or work and specific hormonal exposures.

## Introduction

### Hydraulic fracturing

**Figure 1:**
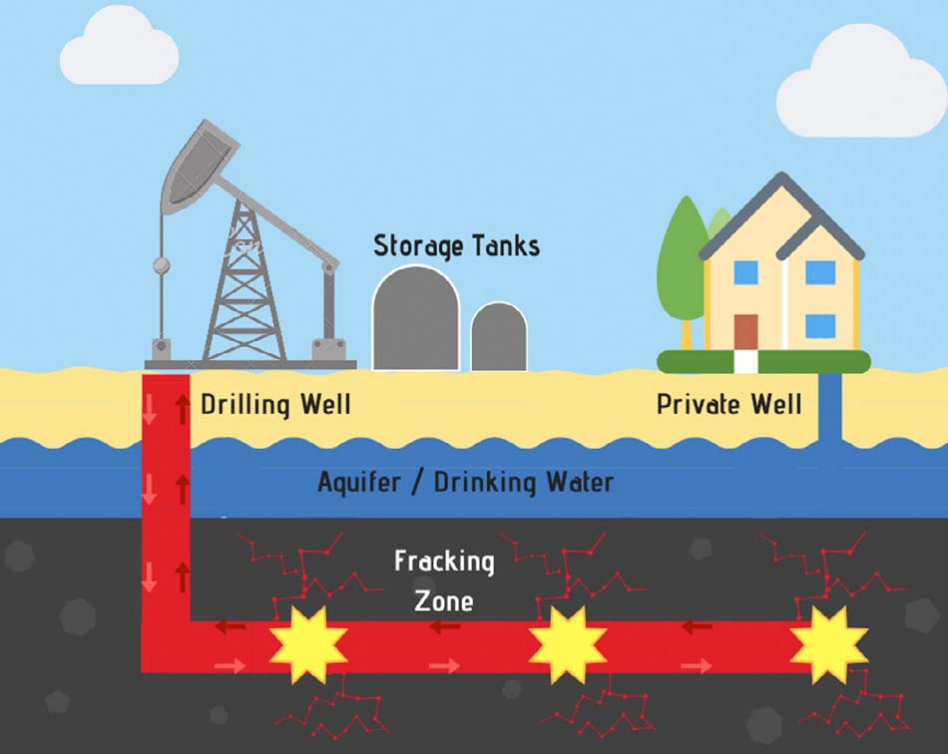
Schematic detailing the hydraulic fracturing process. The well is drilled vertically downward to a certain depth up to 5000 ft (reported as the well drill depth) and then horizontally radiating out by 1–2 miles (not reported by FracFocus). The fracking occurs in the horizontal space after perforating the casing and delivering proppant (sand or silica plus hydraulic fracturing fluid) to keep the fissures open releasing the oil or gas kept in the shale within the earth.

Hydraulic fracturing is a technique used to release the oil or gas held within naturally occurring pockets of shale or other dense rock often contained deep within the earth (Figure 1) (*1*). While the first patents related to the hydraulic fracturing process date back to 1968 (*2*), a major increase in hydraulic fracturing or ‘fracking’ as it is commonly called occurred starting in the early 2000s, with some reporting a 10-fold increase between 2000 and 2015 (*3**,**4*).

Following this explosion of growth in the hydraulic fracturing industry, initial reports began to suggest that earthquakes were correlated with fracking activity (*5*). These reports were later followed up by numerous studies pointing to increases in seismic activity that correlated with hydraulic fracturing fluid injections (*6*) that are related to the underlying fault lines that these injection wells are perturbing (*7*).

### Implications of hydraulic fracturing fluid on human health

Important health implications exist for those living near hydraulic fracturing sites. Close proximity to hydraulic fracturing sites has been linked with increased hospital utilization (*8*), increased risk of preterm birth (*9*) and increases in congenital heart defects and possibly neural tube defects (*10*). These adverse health outcomes are likely due to the chemicals used in the fracking process. These chemical mixtures are known to affect processes involving development and reproduction (*11*). Therefore, knowing the specific chemicals used in hydraulic fracturing sites near a person’s home and whether they regulate various hormonal pathways, including testosterone and/or estrogen, is important for both researchers who may be studying health outcomes among these populations, but also for those living in the potentially affected communities who may be able to take action (e.g. water testing).

### Communities seek location-based information regarding potential exposure

Hydraulic fracturing locations are important for researchers, clinicians and individuals to be aware of due to the potentially important implications that fracturing sites have on private well water quality (*12*). Methane contamination has been reported in drinking water wells located in close proximity to hydraulic fracturing well sites (*13*). Others found that increased stray gas was found in drinking water wells in close proximity to hydraulic fracturing sites (*14*). Collectively, this research indicates that more rigorous water testing is needed for homeowners using private water wells living in close proximity to hydraulic fracturing sites. Some community-based methods have been employed to test private water wells in close proximity to hydraulic fracturing sites, but in many of these cases, the proportion of contaminants found in wells was low (*15*).

### Access to information about hydraulic fracturing locations and the hormonal mechanisms of contaminants is a major need for communities

The majority of USA individuals is undecided with regards to hydraulic fracturing, in large part because they do not know much about it (*16*). In addition, hydraulic fracturing is a divisive topic among communities (*17*), in part due to the lack of concrete knowledge related to health outcomes. The debate regarding if there are social benefits of hydraulic fracturing in communities (e.g. increased accessibility to higher-income employment) is ongoing. A recent study found that environmental injustice is occurring among some communities where the health risks take place without corresponding financial gain (*18*). However, these issues lead to complex difficulties among communities that seek to reconcile the social and scientific assessments of the risk of hydraulic fracturing (*19*).

### Overview of WellExplorer

The purpose of WellExplorer is 2-fold: (i) to integrate information on hormonal, estrogen and testosterone pathways and the proteins that are targeted by chemicals used in hydraulic fracturing fluid to enable both researchers and community members to readily access this information when studying health outcomes and (ii) to include information about well locations in an easy-to-use manner so that community members can search their own zip codes to locate hydraulic fracturing wells in close proximity to them (ranked by distance from entered zip code). Therefore, WellExplorer (http://www.wellexplorer.org/) is useful to both researchers studying health outcomes related to proximity to hydraulic fracturing wells and also to community members deciding what chemicals they should test for in their private water wells.

## Methods

A diagram illustrating the hydraulic fracturing process is given in Figure 1. This diagram details how the hydraulic fracturing process works and potential routes whereby hydraulic fracturing fluid may enter into private drinking wells (i.e. through the cracks generated via the ‘fracking’ process).

### Cleaning FracFocus and linking with T3DB

The FracFocus online database offers several files available for download that contain up to 45 fields about hydraulic fracturing wells across the USA. FracFocus.org serves as a central registry for hydraulic fracturing chemical disclosures in the USA (*24*). However, it does not contain information regarding the biological action of the chemicals used in the fluids. Our first step was to download and clean the dataset. The dataset consisted of 134 900 unique wells. Wells typically use more than one ingredient in their hydraulic fracturing mixture and therefore in total there were 3.3 million entries where one entry constituted a specific ingredient used at a given well (a well-ingredient pair). We used a subset of fields available from FracFocus to include in WellExplorer—namely, well name, operator name, state name, county name, latitude, longitude, ingredient name, chemical abstract service (CAS) number, supplier, ingredient purpose, total vertical depth and total base water volume.

**Figure 2:**
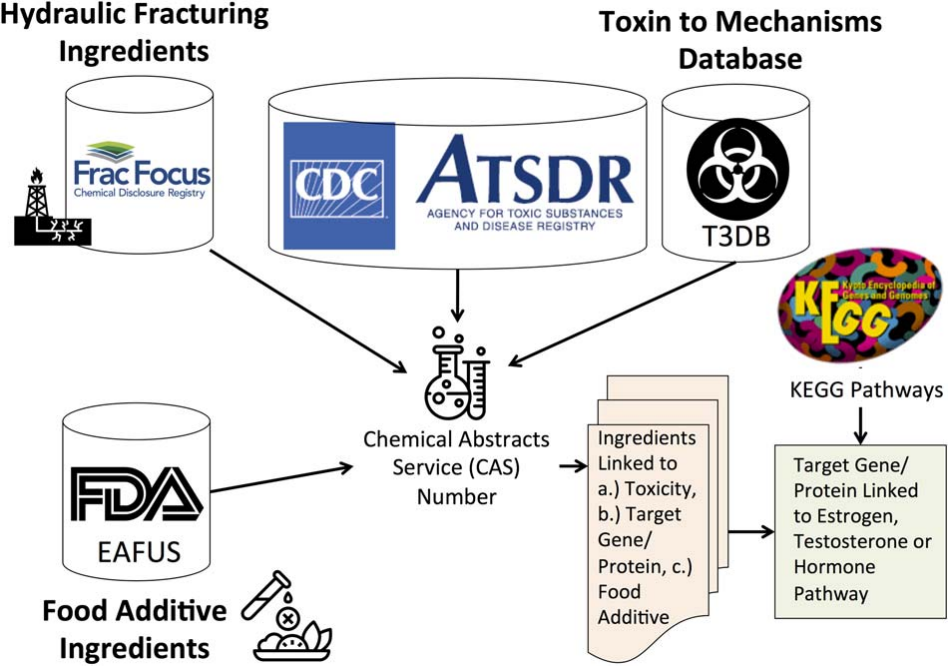
Method for linking hydraulic fracturing ingredients (i.e. chemicals) with information on toxicity (ATSDR), gene and protein targets (T3DB) and food additives Everything Added to Food in USA (EAFUS). We linked the hydraulic fracturing ingredients to information on toxicity, gene and protein targets for each chemical and whether or not the chemical was a food additive using their Chemical Abstracts Service (CAS) numbers. We further linked the gene/proteins to estrogen, testosterone or hormone pathways using KEGG pathways.

Because we also wanted to provide toxic and biological properties of the ingredients found at these well sites, in addition to this FracFocus data, we integrated data from the T3DB database. Using the list of CAS numbers from the cleaned FracFocus data, we linked these well ingredients with the T3DB toxin database information. For those ingredients listed in T3DB, we were able to obtain information on chemicals’ protein targets (and the genes that encode those proteins), toxin mechanisms of actions and specific protein functions. Furthermore, we extracted the toxicity rankings of the top 275 most toxic ingredients from the Agency for Toxic Substances and Disease Registry (ATSDR), as well as a list of ingredients that were food additives as described by Substances Added to Food Inventory, managed by the Federal Drug Administration (FDA). Our overall linkage method is shown in Figure 2.

### Connecting chemical ingredients used in hydraulic fracturing fluid with hormonal pathways

Once we had derived this cleaned and linked dataset of hydraulic fracturing chemicals from the aforementioned data sources, we looked to expand and simplify this data by identifying and annotating ingredients that affected certain hormonal pathways, and the proteins those ingredients targeted. To do this, we looked at the specific function, full gene name (for proteins targeted by the chemicals) and gene synonyms of all the targets in the T3DB dataset and if they involved interactions with ‘estrogen’, ‘testosterone’ or another ‘hormone’. Specifically, we looked to see if any of the targets in T3DB explicitly contained these three respective words in one of those fields (function, full gene name and gene synonyms), all genes falling into this category being subsequently labeled ‘estrogen gene’, ‘testosterone gene’ and/or ‘hormone gene’, depending on which of the three words their field(s) contained. To further identify genes involved in hormonal pathways, we used all of the Kyoto Encylopedia of Genes and Genomes (KEGG) pathways that contained an estrogen, testosterone and/or hormone gene and labeled those as ‘estrogen’, ‘testosterone’ or ‘hormone’ pathways. Therefore, for example, we labeled an ingredient as hitting an estrogen pathway if that ingredient targeted any of the genes/proteins in the same pathway. This labeling resulted in six new binary fields: estrogen gene, testosterone gene, hormone gene, estrogen pathway, testosterone pathway and hormone pathway. A diagram illustrating how T3DB annotated protein/gene information was labeled as belonging to estrogen, testosterone, hormone genes and their corresponding pathways is shown in **Supplemental Figure S1****.**

### WellExplorer interface functionality

Because part of our goal in cleaning and correlating this data was to make our conclusions and final data easily accessible to not only researchers but also to the layperson, we wanted to create a web interface through which these cleaned datasets could be easily browsed and interpreted. Since there are already sites that present in-depth hydraulic fracturing well data in an accessible way, we looked to specialize our interface more toward the unique aspects of the data analysis that we performed (as detailed above), particularly the biological and toxic properties of the ingredients contained inside these wells. In this way, we looked to include specific information about the proteins these ingredients target and the ultimate hormones and hormonal pathways that could be affected, as well as useful links regarding the ingredients and genes themselves. These genes encode proteins targeted by the chemicals.

Well proximity is one of the most important and relevant considerations when discussing the potential effect these ingredients could have on an individual’s exposome; we also looked to include a Zip Searcher, or position locator function in the WellExplorer application. Specifically, we wanted the browser to not only be able to display wells close to the entered location but to also list the distances of these wells from the input position and to return them by absolute proximity to the location. Finally, we included options to narrow and shorten the number of well results by querying the data by its different fields, including well name/operator name, ingredient name/CAS number and affected gene name/affected pathways.

### WellExplorer interface development and implementation

In terms of developing this web application, we first cleaned, shortened and subsetted the data to create two newly compressed, usable and formatted .csv files that could be efficiently used in the deployment of the browsing site, ultimately dubbed WellExplorer. The first of these files is well_data, which contains a list of the wells and ingredients, which we aggregated by well name for ease of interpretation, with general well information as well as ingredient details that are specific to that well, such as supplier and ingredient purpose. The second is ingr_path_props, which consists of the more ingredient-specific data, as well as target proteins (and the genes encoding those proteins), and pathway-related information; we aggregated this dataset by ingredient name, with each ingredient having a corresponding list of targets.

To develop the Zip Searcher function, we utilized the Geocoding Google Maps API to extract the latitude and longitude from the entered location. This position is then passed, in real time, to the Haversine Distance Formula along with the well latitude and longitude positions, to compute the absolute distances between the entered location and the well sites. The haversine distance formula is appropriate here because of its accuracy in calculating distances on a curved surface, such as the earth.

We also developed the Data Explorer, a platform through which users can more directly interact, query and download these two datasets. To create this, we used the DataTables package in conjunction with the R Shiny package, which was the library ultimately used to develop the entire web application, both of which are offered freely by R Studio. Our initial version used comma separated (.csv) files linked together in our R script. Additionally, to deal with information bias, data warning flags were set for any well where the data seemed to be inaccurate, e.g. when the depth of the well was reportedly above 40 000 feet (which is the depth of the largest known well in the world), or the longitude and latitude do not fall within the stated county name. Finally, we created well landing page that contained much more in-depth information about the well itself and all of the relevant details about the ingredients used at the well. This included information regarding suppliers, toxicities, protein targets and pathways. We also linked the latitude and longitude positions of the well to Google Maps, so that the position of the wells could be seen more visually. In addition, we used the T3DB and UniProt IDs to link the ingredients and proteins to t3db.ca and uniprot.org, providing even more information about certain ingredients and proteins.

## Results

### Overview of WellExplorer data

We cleaned the data from FracFocus and found that 1276 unique chemicals with CAS numbers were listed as ingredients in hydraulic fracturing fluids. We also found that 29 non-blank unique ingredients were not mapped to CAS numbers. Most of these non-CAS mappable chemicals contained the name ‘other’ in them (e.g. ‘other inorganic magnesium compound’) or were non-specific (e.g. ‘organic amine resin salt’) or were vague (e.g. BE-9). We linked the 1276 unique chemicals with CAS numbers to our information on hormonal pathway, estrogen pathway and testosterone pathways obtained by linking with KEGG (Figure 2) with illustrative example shown in **Supplemental Figure S1**. The list of genes and pathway IDs from T3DB that were annotated as belonging to estrogen, testosterone or hormone pathways is given in **Supplemental Table S1**. We found that 34 unique ingredients target proteins in the estrogen pathway, 8 unique ingredients target proteins in the testosterone pathway and 51 unique ingredients target hormonal pathways (e.g. insulin or thyroid hormone pathways). The chemicals targeting these various pathways are given in **Supplemental Table S1****.**

### 3.2. Geographic differences among hormonal targets of hydraulic fracturing fluids

**Figure 3:**
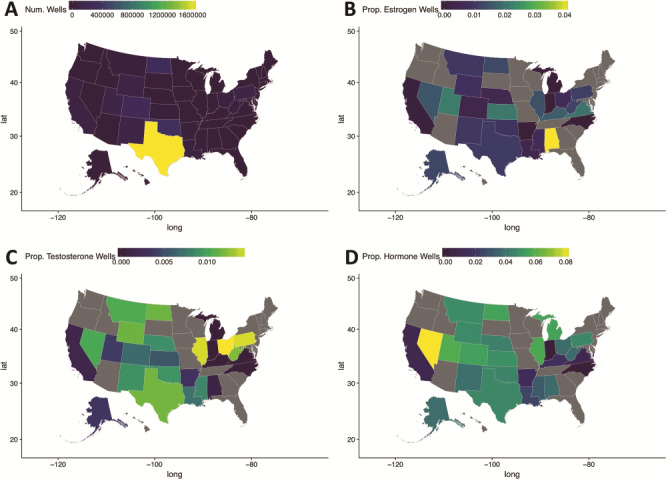
Maps of the United States of America detailing hydraulic fracturing well locations (**A**), the proportion of wells with estrogen pathway-targeting Ingredients (**B**), the proportion of wells with testosterone pathway-targeting Ingredients (**C**) and the proportion of wells with hormonal pathway-targeting ingredients (**D**). Notice that states with elevated proportions of certain estrogen, testosterone or hormonal pathway ingredients relative to other hydraulic fracturing wells can be easily identified using these visual maps.

Linking the hydraulic fracturing wells to their hormonal modulators using the method outlined in Figure 2 enabled us to visualize areas in the country with relatively high proportions of hydraulic fracturing wells that were utilizing ingredients affecting various hormones. We found that Alabama has a high proportion of wells using ingredients that target estrogen pathways (Figure 3). Whereas, Illinois, Ohio and Pennsylvania have a high proportion of hydraulic fracturing wells that were utilizing ingredients that target testosterone pathways (Figure 3). We also found that Nevada had a high proportion of wells that were targeting hormonal pathways in general (e.g. thyroid hormone pathways) (Figure 3).

### Interacting with WellExplorer for community members

**Figure 4:**
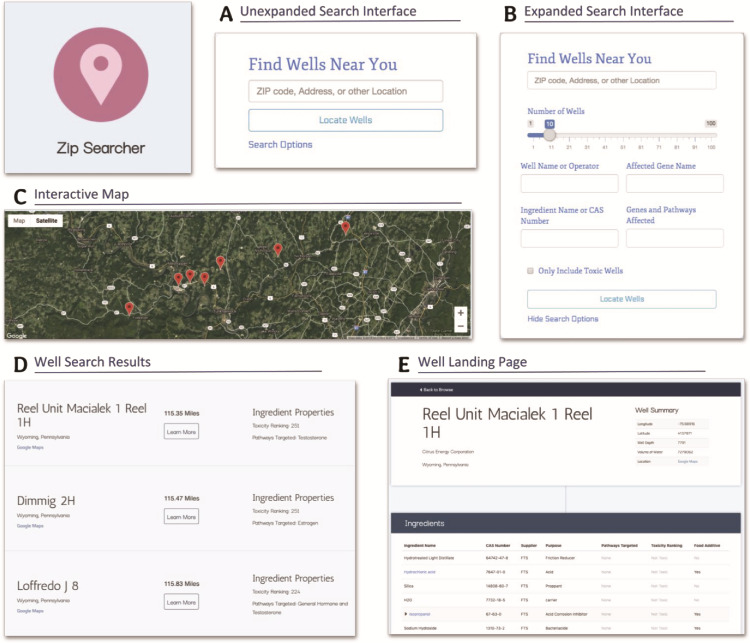
Screenshots from Zip Searcher and well landing pages detailing the different functionalities, fields and information contained on these pages. (**A**) The simple, collapsed search interface. (**B**) The more detailed, expanded search interface. (**C**) The interactive map displayed at the top of the results list. (**D**) The well search results containing the well and operator name, a summary of the well’s ingredient properties, the absolute distance from the entered location to the well, and a ‘learn more’ button. (**E**) The well landing page, displaying more in-depth information about the well as well as a comprehensive list of the well’s ingredients and the properties of these ingredients. We made these pages specifically with interpretability in mind—the search fields are easy to understand, and most of the information displayed is self-explanatory and does not require much background knowledge to interpret.

Because WellExplorer has two purposes: one for researchers and the second for interested community members, we constructed the interface to have several features that are specifically designed to be used and interpreted by community members. The Zip Searcher page, in particular, was developed with community members in mind. As shown in Figure 4A, the initial search interface landing page is simplistic, only containing the required location field, allowing community members to quickly and easily access local well data by either entering their zip code, address or location (e.g. Blockley Hall) that is compatible with Google Maps. The expanded search interface (Figure 4B), while containing several more technical fields, does additionally offer some easily interpretable fields such as ‘well name or operator’ and ‘only include toxic wells’ that can help the user obtain more applicable results without requiring them to have deep background knowledge regarding toxicity and biological pathways.

Furthermore, the results themselves are presented in a readable manner. The first result that is displayed to the user (after they search a location) is an embedded interactive map that displays the first 10 wells on a satellite map (Figure 4C). The well results themselves are intended to be understandable, and therefore, only more generalized information is initially displayed with options and buttons to learn more about the specific data shown. This is displayed in Figure 4D, which displays three sample well results containing well and operator name information, simplified ingredient properties, the absolute distance from the well to the center of the entered location, and a ‘learn more’ button that links to that specific well’s landing page. While the information contained in this well result (in addition to its visual representation on the interactive map) is a broad overview of the information needed by community members, more detailed information regarding a particular well is available if a user navigates to the landing page of that well (by clicking the ‘learn more’ button) to view more in-depth well information as well as a detailed list of the specific properties of each ingredient used at that well site (Figure 4E).

### Interacting with WellExplorer for researchers

**Figure 5:**
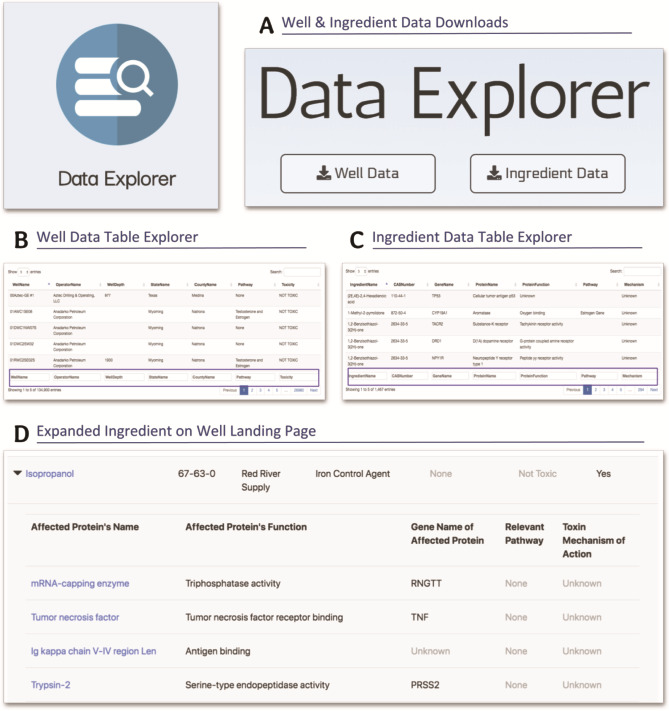
Screenshots from the Data Explorer and well landing pages detailing the download capabilities, data tables and specific, in-depth information available on these pages. (**A**) The data download buttons for the well and ingredient data. (**B**) The well data table explorer with search and sort functionality. (**C**) The ingredient data table explorer with search and sort functionality. (**D**) The expanded isopropanol ingredient on the well landing page, displaying the genes, proteins and biological pathways affected by this ingredient. We created the Data Explorer page and the expanded ingredients feature of the well landing page with a more research-purposed and knowledgeable audience in mind.

Additionally, WellExplorer also caters to researchers and academics. A Data Explorer page was specially designed with researchers in mind and it contains advanced functionality. As shown in Figure 5A, the Data Explorer page includes options to download either the well data, which powers the well information and distance calculations, or the ingredient data, which contains the toxicity and pathway-related information. Those interested in doing further research into the regional effects of hydraulic fracturing wells can use these freely accessible and cleaned data sets to power their research. The Data Explorer Page contains both well and ingredient data tables (**Figure 5A** and **B**) for viewing the raw data that can be downloaded for additional research purposes. Not only are these data tables very comprehensive, having 134 900 unique wells and 1467 unique ingredients, they also have a variety of different filtering and sorting features that can allow increased accessibility. Specifically, each data table can be sorted, alphabetically or numerically, depending on the data type, by each individual field. In addition, the search bars at the bottom of each column (highlighted with purple outline in **Figure 5B** and **C**) allow the user to filter the data by each of these individual fields. This filter-by-field feature is especially applicable to researchers concerned with the proteins (and the genes that encode them) affected by these potentially toxic ingredients by allowing them to filter the ingredients in accordance with their particular gene/protein of interest.

The Zip Searcher page also has some advanced functionality for researchers. In particular, the expanded search interface (Figure 4B) allows users to enter specific gene names and pathways (hormone, estrogen testosterone) that the ingredients used by a particular well could affect. The Zip Searcher then takes this input and first filters the data based on these genes and pathways before sorting and displaying the well results by absolute distance. This could help researchers not only see which wells have ingredients involved in certain biological pathways but also the geographic position of these specific wells, perhaps aiding in the identification of regions with a higher exposome potential in regards to a certain gene or pathway.

The well landing page also contains some more advanced functionality that could be utilized by a researcher—namely, in the ingredients section. Not only does each ingredient contain a plethora of information about the general properties of that ingredient (Figure 4E)—specifically ingredient name, CAS number, supplier, purpose, pathways targeted, toxicity ranking and food additive—some ingredients can also be expanded to reveal more information about the ingredient. Whether or not a specific ingredient has this additional functionality depends on if its CAS number can be found in the T3DB toxin and toxicant database that was used for the linking of this well information to the biological and toxicity information, something that is only true for a handful of ingredients for each well. These certain ingredients are linked to the T3DB online interface, so clicking them will lead you to their ingredient page on the T3DB website (t3db.ca), which contains additional information on the ingredient itself and its toxic properties. In addition, these certain ingredients can be expanded (Figure 4D) to reveal a list of the proteins (and genes) affected by the ingredient—which are also linked, this time to the UniProt website (uniprot.org)—as well as some other fields specific to each of these affected proteins. This functionality of linking this well data to the toxin information is one of the hallmarks of WellExplorer, and it sets it apart from other interfaces.

**Figure 6:**
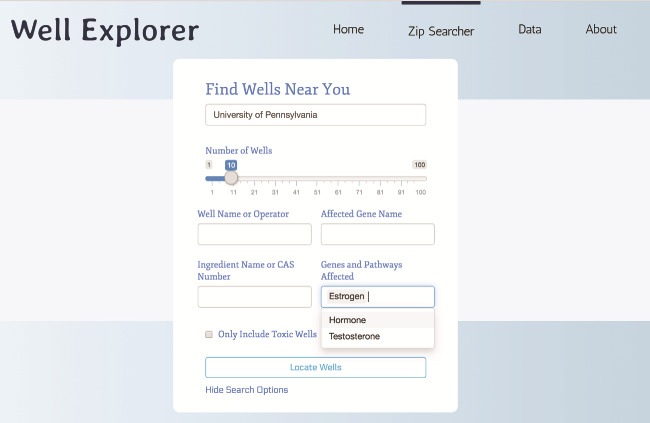
Searching WellExplorer for hydraulic fracturing wells using ingredients that target estrogen pathways. Note that ‘estrogen’ is selected in the ‘genes and pathways affected’ option. Clicking on ‘locate wells’ will retrieve all wells using ingredients that target estrogen pathways ranked from the closest in distance (in miles).

Users can search for hydraulic fracturing wells that are using ingredients targeting estrogen (or testosterone or other hormones) by selecting estrogen in the ‘genes and pathways affected’ option in the dropdown (Figure 6). This will retrieve the closest 10 wells ordered by absolute distance from the location entered (‘University of Pennsylvania’) using ingredients that target the estrogen pathway. This can be useful for both researchers interested in understanding whether or not reproductive outcomes are likely given the proximity of an individual to these wells or this could be used by community members interested in conducting more detailed analyses of their own private water wells.

## Discussion

Hydraulic fracturing fluids contain many diverse chemical ingredients. Elliot *et al*. compared 1021 chemicals used in hydraulic fracturing fluid to REPROTOX (a database containing information on reproductive toxins), and they found that 43% of chemicals with known toxicity information were reproductive toxins (*20*). However, we took a different approach. We show states in the USA that are using a disproportionately large amount of ingredients that target certain hormonal pathways (Figure 3**)**. For example, Alabama has a disproportionately large amount of hydraulic fracturing wells that are using ingredients that target estrogen pathways. Whereas, Ohio, Pennsylvania and Illinois have hydraulic fracturing wells that are using a disproportionately large amount of ingredients that target testosterone pathways. On the other hand, Nevada has a disproportionately large amount of hydraulic fracturing wells that are targeting hormonal pathways in general. This information can be important for researchers studying exposure to hydraulic fracturing fluids (*21*) and community members interested in knowing what they should be testing for in their private well water.

Currie *et al*. demonstrated a link between being born a low-birth weight baby (an adverse infant outcome) and close proximity to hydraulic fracturing wells in Pennsylvania (*21*). It is possible that this is due to the ingredients that are being used in Pennsylvania at disproportionately target testosterone pathways (Figure 3). It is also possible that, in other states where those ingredients are used to a lesser extent, the infant outcomes may differ. This could also explain the difficulties that researchers have had with studying hydraulic fracturing fluids and linking exposure with health outcomes (i.e. the diversity of ingredients used in different locations across the USA) (*22*). This diversity in type of exposure is also the likely reason why broad USA-wide health outcomes studies for exposure to hydraulic fracturing fluid remain lacking (*23*).

A major contribution of WellExplorer (http://www.wellexplorer.org/) is the ease with which regular community members and individuals can query and interact with WellExplorer to learn more about exposures near them or their loved ones. All prior health outcomes studies have used pre-existing health data to link with proximity to hydraulic fracturing fluids. However, these studies do not provide individuals in the community with the information that they need to understand their own health risk. By creating WellExplorer, we have made a tool that can be useful to researchers to understand the proteins that these hydraulic fracturing fluid ingredients target and the hormonal implications of this targeting, including the genes encoding these protein targets. In addition, we have made a tool that community members can utilize to learn more about the areas where they live and the potential for inadvertent exposure to hormonal modulators in their communities. By providing community members with the information in WellExplorer, we are disseminating scientific research back to those whom are the most impacted by its results. Furthermore, we are providing a sense of autonomy and control to environmental justice communities, disproportionately burdened by fracking. With WellExplorer, concerned citizens can take note of the chemicals that are found nearest to their water source and test their wells or drinking water for these chemicals. It provides them with a scientific basis for them to then make decisions from. These decisions include not only testing for chemicals but also decisions about whether they will drink their water, which can be critical to the health of families. A lack of feeling in control breeds fear and uncertainty; WellExplorer provides an actionable resource to vulnerable communities.

A major limitation of this work is the current state of the art knowledge with regards to the ingredients used in hydraulic fracturing fluid. We only were able to link ingredients with valid CAS numbers that were also found in T3DB with regards to their potential for toxicity. T3DB also linked the CAS number with genes/proteins that the CAS ingredient target. We were able to use the gene/protein information to link to information on the estrogen, testosterone or hormonal pathways using KEGG. However, all of these resources are still be updated as current research is published, and more information is made available about these CAS ingredients used in hydraulic fracturing fluid. We intend on updating WellExplorer twice per year to ensure that it remains updated with the latest current state of the art with regards to this information. However, this is still an evolving field.

In conclusion, we have developed WellExplorer (http://www.wellexplorer.org/), which contains information on hydraulic fracturing wells and also detailed information on the ingredients used in the hydraulic fracturing fluid. This includes whether the ingredient is a toxin, a food additive, known to target a hormonal pathway, known to target an estrogen pathway, or known to target a testosterone pathway. We also include specific information on the gene targeted by the ingredient, if that information is available. Use of WellExplorer enables two ends: (i) to integrate information on hormonal, estrogen and testosterone pathways and genes that hydraulic fracturing fluid ingredients target so that researchers and community members can readily access this information when studying health outcomes and (ii) to include information about well locations in an easy-to-use manner so that community members can search their own zip codes to locate hydraulic fracturing wells in close proximity to them (ranked by distance from entered zip code). WellExplorer is useful to both researchers studying health outcomes related to proximity to hydraulic fracturing wells and also to community members deciding what chemicals they should test for in their private water wells. Researchers using WellExplorer can also search for specific gene names that they may be interested in to locate wells that are using ingredients targeting those genes. This could be very important for future research questions investigating health outcomes following exposure to hydraulic fracturing fluids.

## Supplementary data

Supplementary data are available at *Database* online.

## Supplementary Material

Hydraulic_Fracturing_Paper_Supplemental_Database
