# Supplementary material for: WellExplorer: an integrative resource linking hydraulic fracturing chemicals with hormonal pathways and geographic location
Source: Database (Oxford). 2020 Jul 23;2020:baaa053. doi: 10.1093/database/baaa053 (PMC7376760; doi:10.1093/database/baaa053)
Supplement: Hydraulic_Fracturing_Paper_Supplemental_Database [file hydraulic_fracturing_paper_supplemental_database.pdf]

Supplemental Figures and Tables for:
Wetherbee O, Meeker J, DeVoto C, Penning T, Moore J, Boland MR.
WellExplorer: An Integrative Resource Linking Hydraulic Fracturing Chemicals with Hormonal Pathways and Geographic Location

Table of Contents

Tables

Table S1. Hydraulic Fracturing Ingredients Targeting Estrogen, Testosterone or Hormone Pathways..................2

Figures

Figure S1. Illustrative Diagram Detailing the Method of Linking Genes to Estrogen, Hormone and Testosterone Genes and Pathways...4

SUPPLEMENTAL MATERIAL

Table S1. Hydraulic Fracturing Ingredients Targeting Estrogen, Testosterone or Hormone Pathways

CAS Number	Chemical Name
**Chemicals Targeting Estrogen Pathways**	
101-77-9	Benzenamine, 4,4'-methylenebis-
104-40-5	Nonyl Phenol, 4 Mol
106-24-1	2,6-Octadien-1-ol, 3,7-dimethyl-, (2E)-
107-02-8	Acrolein
107-98-2	1-Methoxy-2-Propanol
108-38-3	Benzene, 1,3-dimethyl-(Xylene)
108-83-8	Disobutyl Ketone
110-17-8	Fumaric Acid
111-42-2	Diethanolamine
111-44-4	Dichloroethyl ether (DCEE)
117-81-7	Di (2ethylhexyl) Phthalate
126-92-1	Sodium-2-ethylhexyl sulfate
1344-28-1	Aluminum Oxide
138-22-7	Butyl lactate
139-13-9	Nitriloacetic Acid
151-21-3	Lauryl Sulfate
1762-95-4	Ammonium Thiocynate
25322-69-4	Polypropylene Glycol
486-66-8	Soy Isoflavone
50-28-2	Silica Substrate
52-51-7	2-Bromo-2-Nitro-1,3-Propanediol
62-56-6	Thiourea
65-85-0	Benzoic Acid
7429-90-5	Aluminum Needles
7440-02-0	Nickel
7446-70-0	Aluminum Chloride
7646-79-9	Cobalt chloride(CoCl2)
7664-38-2	Phosphoric Acid
7718-54-9	Nickel(II) Chloride
7786-81-4	Nickel Sulfate
78-40-0	Triethyl Phosphate
872-50-4	N-Methyl Pyrrolidone
9004-82-4	Alpha-Sulfo-w-(dodecyloxy)-poly(oxyethane-1,2-diyl), sodium salt
92-52-4	BIPHENYL
**Chemicals Targeting Testosterone Pathways**	
10025-91-9	Antimony trichloride
107-98-2	1-Methoxy-2-Propanol
111-30-8	Glutaraldehyde
111-40-0	Diethylenetriamine
25038-59-9	Polyethyleneterephthalate
50-28-2	Silica Substrate
533-74-4	3,5-Dimethyltetrahydro-1,3,5-thiadiazine-2-thione
8001-54-5	Quaternary ammonium compounds, alkylbenzyldimethyl, chlorides
**Chemicals Targeting Hormone Pathways**	
10025-91-9	Antimony trichloride
101-77-9	Benzenamine, 4,4'-methylenebis-
104-40-5	Nonyl Phenol, 4 Mol
106-24-1	2,6-Octadien-1-ol, 3,7-dimethyl-,(2E)-

107-98-2	1-Methoxy-2-Propanol
108-38-3	Benzene, 1,3-dimethyl-(Xylene)
108-83-8	Disobutyl Ketone
111-30-8	Glutaraldehyde
111-40-0	Diethylenetriamine
111-42-2	Diethanolamine
111-44-4	Dichloroethyl ether (DCEE)
112-80-1	Oleic Acid
117-81-7	Di (2ethylhexyl) Phthalate
1184-78-7	Trimethylamine, N-oxide
126-92-1	Sodium-2-ethylhexyl sulfate
128-37-0	Butylated hydroxytoluene
1344-28-1	Aluminum Oxide
138-22-7	Butyl lactate
139-13-9	Nitriloacetic Acid
151-21-3	Lauryl Sulfate
1762-95-4	Ammonium Thiocynate
25038-59-9	Polyethyleneterephthalate
2634-33-5	1,2-Benzisothiazolin-3-one
486-66-8	Soy Isoflavone
50-28-2	Silica Substrate
52-51-7	2-Bromo-2-nitro-1,3-propanediol
533-74-4	3,5-Dimethyltetrahydro-1,3,5-thiadiazine-2-thione
56-86-0	L-Glutamic acid
62-56-6	Thiourea
64-17-5	Ethyl Alcohol
64-19-7	Acetic Acid
65-85-0	Benzoic Acid
71-43-2	Benzene
71-48-7	Cobalt Acetate
7429-90-5	Aluminum Needles
7440-02-0	Nickel
7446-70-0	Aluminium Chloride
7487-88-9	Magnesium Sulfate
75-50-3	Trimethylamine
7646-79-9	Cobalt chloride(CoCl2)
7664-38-2	Phosphoric Acid
7664-41-7	Ammonia
7718-54-9	Nickel(II) Chloride
7772-99-8	Stannous Chloride Dihydrate
7786-81-4	Nickel Sulfate
78-40-0	Triethyl Phosphate
80-08-0	4,4`-Diaminodiphenyl sulfone
8001-54-5	Quaternary ammonium compounds, alkylbenzyldimethyl, chlorides
9004-82-4	Alpha-Sulfo-w-(dodecyloxy)-poly(oxyethane-1,2-diyl), sodium salt
91-20-3	Naphthalene
92-52-4	BIPHENYL

Gene Name	Full Gene Name	Specific Function	Gene Synonyms	KEGG ID	Estrogen Gene	Hormone Gene	Testosterone Gene
ESR1	**Estrogen** receptor	Nuclear **hormone** receptor…	ER; ER-alpha; ESR; Estradiol receptor; NR3A1; Nuclear receptor subfamily 3 group A member 1	hsa:2099	**Yes**	**Yes**	No
HSD17B1	Estradiol 17-beta-dehydrogenase 1	Favors the reduction of **estrogens** and androgens…	1.1.1.62; 17-beta-HSD 1; 17-beta-hydroxysteroid dehydrogenase type 1…	hsa:3292	**Yes**	No	No
HSD3B1	3 beta-hydroxysteroid dehydrogenase/Delta 5-->4-isomerase type 1	3-beta-HSD is a bifunctional enzyme… dihydro**testosterone** to 5-alpha-androstane-3 beta,17 beta-diol…	3 beta-hydroxysteroid dehydrogenase/ Delta 5-->4-isomerase type I; 3-beta-HSD I; 3BH; HSDB3A; Trophoblast antigen FDO161G	hsa:3283	No	No	**Yes**
JAK2	Tyrosine-protein kinase JAK2	…plays a pivotal role in signal transduction via its association with type I receptors such as growth **hormone** (GHR), prolactin (PRLR)…	2.7.10.2; JAK-2; Janus kinase 2	hsa:3717	No	**Yes**	No

KEGG ID Used to Identify Other Genes in Same Pathway

Figure S1. Illustrative Diagram Detailing the Method of Linking Genes to Estrogen, Hormone and Testosterone Genes and Pathways. The pathway linkage was done using the KEGG IDs. For example, JAK2 is labeled as a hormone gene with corresponding KEGG ID (hsa:3717) any other genes in T3DB containing the same KEGG ID (hsa:3717) would be labeled as belonging to the hormone pathway even if the word 'hormone' was not explictly found in the name, specific function or synonym fields.
